# Taxonomic implications from morphological and anatomical studies in the section *Stenodiptera* from the genus *Grammosciadium* (Apiaceae)

**DOI:** 10.3897/phytokeys.68.9089

**Published:** 2016-08-09

**Authors:** Barış Bani, Fatma Ulusoy, Muhammet Ali Karakaya, Marcus A. Koch

**Affiliations:** 1 Kastamonu University, Faculty of Arts and Sciences, Department of Biology, 37200, Kuzeykent, Kastamonu, Turkey; 2 Heidelberg University, Centre for Organismal Studies, Department of Biodiversity and Plant Systematics, 69120 Heidelberg, Germany

**Keywords:** Grammosciadium, MANOVA, PCA, Stenodiptera, taxonomy, Turkey

## Abstract

Grammosciadium pterocarpum subsp. bilgilii and Grammosciadium pterocarpum subsp. sivasicum from Turkey are herein described as two new subspecies, and the species *Grammosciadium schischkinii* is synonymied under Grammosciadium pterocarpum subsp. pterocarpum. Quantitative variation of morphological and anatomical characters have been analysed to provide discriminative characters between the taxa of section *Stenodiptera* and to provide a key to the species. The taxonomic status of the taxa has been discussed in light of these morphological and fruit anatomical data using multivariate statistics such as MANOVA and Principal Component Analysis. The results are also used to present a critical discussion of characters used to distinguish and determine different taxa within *Grammosciadium*. MANOVA showed that ten characters, except stylopodium and style length, differed significantly among the taxa, and the results were confirmed by Tukey tests and PCA analysis (except the character of fruit number). However, only ranges of the characters of sepal length, fruit length, fruit width, fruit width/wing width ratio, and width of fruit wing are not overlapped. Qualitative characters of petiolate stipular segments of lower leaves and presence of funicular oil ducts in transvers section of mericarps were found as diagnostic characters.

## Introduction

The genus *Grammosciadium* DC. is a taxonomically difficult group of taxa within tribe Scandiceae, subtribe Careae (Apiaceae) (Spalik et al. 2001, Spalik and Downie 2007, Ajani et al. 2008). It falls within the “apioid superclade” (Spalik and Downie 2007) and is closely related with *Fuernrohria* K.Koch, *Carum* L. and other taxonomically critical taxa. As an example, in particular the genus *Carum* has been recently shown to be polyphyletic in its current circumscription, and its various members are even found in different tribes of subfamily Apioideae (Zakharova et al. 2012). The stem group age of Careae is of about 22 million years and has a center of origin in the Irano-Turanian region (Banasiak et al. 2013), and a long-term and spatio-temporarely shared evolutionary history of the various lineages is likely. Accordingly, taxonomy and systematics of these groups of taxa are still unsolved, because morphological characters often display high levels of homoplasy, and phylogenetic inference is scarce (e.g. Downie et al. 2010, Zakharova et al. 2012).

The genus *Grammosciadium* is actually considered to be represented by two subgenera (*Grammosciadium* and *Caropodium* (Stapf & Wettst.) Tamamsch. & V.M.Vinogr.), which are further split into six sections in total (Bani and Koch 2015); however, this has been done with limited available pyhlogenetic evidence so far and applying an extreme taxanomically splitting concept above the species level. Subgenus *Caropodium* has been further splitted into two sections, namely sects. *Caropodium* and *Stenodiptera* (Koso-Pol.) Tamamsch. & V.M.Vinogr. (for a detailed overview refer to Bani and Koch 2015).

The section *Stenodiptera* of the genus *Grammosciadium* DC. has typical mericarps with winged lateral ribs. Additional important characters are the presence of erect stems, white flowers, and 4-6-pinnatisect, narrowly linear-elliptic leaves. The section *Stenodiptera* morphologically resembles section *Caropodium* because of the winged fruits, which are absent in other members of the genus *Grammosciadium* (Tamamschian and Vinogradova 1969a, b, 1970, Vinogradova 1995). However, the section *Caropodium* mainly differs from section *Stenodiptera* by its more branched and distinctly sulcate stems (Hedge and Lamond 1972).

Both sections are also anatomically different from each other: section *Caropodium* has funicular oil ducts in transvers section of mericarps (funicular oil ducts absent in section *Stenodiptera*) (Tamamschian and Vinogradova 1969a, b, 1970, Vinogradova 1995). Also fruit surface ornamentations is different in both sections (Bani et al. 2016). Since both of these sections are included in subgenus *Caropodium* of genus *Grammosciadium* in the actual literature (Tamamschian and Vinogradova 1969a, b, 1970, Vinogradova 1995), we still follow the concepts of sections and subgenera for pragmatic reasons, but being fully aware that this is an artificially splitting concept and is awaiting phylogenetic analysis (work in progress).

The section *Stenodiptera* has three species which are distributed mainly in Turkey, and are additionally found in adjacent areas of Anatolia and Iran (Hedge and Lamond 1972, Vinogradova 1970, Bani and Koch 2015). All members are characteristic plants of the Irano-Turanian phytogeographic region (Takhtajan 1986, Hedge and Lamond 1972, Tamamschian 1987, Bani and Koch 2015). Among them, *Grammosciadium schischkinii* (V.M.Vinogr. & Tamamsch.) V.M.Vinogr. and *Grammosciadium haussknechtii* Boiss. are endemic to Turkey and the third species, *Grammosciadium pterocarpum* Boiss., can be regarded as subendemic plant with smaller distribution ranges outside Turkey (Vinogradova 1970, Pimenov and Leonov 2004, Pimenov and Sutory 2014).

After the first record of the genus *Grammosciadium* has been provided (Candolle 1829) Boissier (1844, 1872) described the three species *Grammosciadium pterocarpum*, *Grammosciadium haussknechti* and *Grammosciadium platycarpum* Boiss. & Hausskn. in addition to the other members of the genus [*Grammosciadium daucoides* DC., *Grammosciadium aucheri* Boiss. (currently accepted as synonym of *Grammosciadium daucoides*), *Grammosciadium scabridum* Boiss., *Grammosciadium longilobum* Boiss. & Hausskn. (currently accepted as synonym of *Grammosciadium scabridum*), and *Grammosciadium macrodon* Boiss.]. Boissier indicated in his Flora Orientalis, that these three species are different from the other members of the genus by their winged mericarps (Boissier 1872). Later in 1886, *Caropodium* was established as a new genus with a single species (*Caropodium meoides* Stapf and Wettst.) collected by Polak from Iran (Stapf and Wettstein 1886). However Bornmueller (1906) synonymised *Caropodium meoides* under *Grammosciadium platycarpum*. Freyn (1901) published a subspecies of *Grammosciadium pterocarpum* from Turkey, namely subsp. longipes. The respective type material provides only flowering material and no fruits are available, and, therefore, this taxon is currently accepted as synonym of *Grammosciadium pterocarpum* (Pimenov and Sutory 2014). Koso-Poliansky established *Stenodiptera* Koso-Pol. with all these three winged species as an independent genus (Koso-Poliansky 1914, 1915). Moreover he divided his genus *Stenodiptera* into two sections with *Euryptera* including *Stenodiptera pterocarpa* (Boiss.) Koso-Pol. and *Eustenodiptera* including *Stenodiptera haussknechtii* (Boiss.) Koso-Pol. and *Stenodiptera platycarpa* (Boiss. & Hausskn.) Koso-Pol., which is mostly based on breadth of wings of mericarps (Koso-Poliansky 1914, 1915). Although a species named *Stenodiptera armena* Bordz., which was collected from Turkey, was published by Bordzilowski (1915), Koso-Poliansky (1916) synonymised this species under *Stenodiptera haussknechtii* one year later. According to Schischkin (1923) Koso-Poliansky’s idea of establishing a new genus with the winged members was appropriate, but he added and highlighted an important taxonomic aspect: if a new genus has been established with winged fruited species separated from genus *Grammosciadium* sensu Boissier, then this must be with the earlier published name *Caropodium* rather than *Stenodiptera*. Hence, he reduced the genus *Stenodiptera* into synonymy of the genus *Caropodium* and also re-established *Caropodium armenum* (Bordz.) Schischkin on species rank (Schischkin 1923). Vinogradova and Tamamschian (1968) accepted that “*Caropodium armenum* is identical to *Caropodium pterocarpum* (Boiss.) Schischkin” and they also described Caropodium pterocarpum var. schischkinii V.M.Vinogr. & Tamamsch. as a new taxon based on a specimen collected from Turkey in 1916. This variety was distinguished by its broader and more undulated wings of the fruits (Vinogradova and Tamamschian 1968). Later Tamamschian and Vinogradova (1969b and 1970) reduced *Caropodium* to the rank of a subgenus and recognized section *Stenodiptera* including the taxa *Grammosciadium pterocarpum*, Grammosciadium pterocarpum var. schischkinii and *Grammosciadium haussknechtii* within this subgenus. In Flora of Turkey, Hedge and Lamond (1972) presented Grammosciadium pterocarpum var. schischkinii and *Grammosciadium haussknechtii* as synonyms of *Grammosciadium pterocarpum*. Finally, Vinogradova (1995) increased *Grammosciadium schischkinii* to species rank and also *Grammosciadium haussknectii* was accepted as independent species again.

In summary, there are five taxa in section *Stenodiptera* that have been described so far: *Grammosciadium pterocarpum*, *Grammosciadium haussknechtii*, Grammosciadium pterocarpum subsp. longipes Freyn, *Grammosciadium armenum* and *Grammosciadium schisckinii*. However, for more than a whole century botanists are wondering of how to distinguish and how to classify them.

The taxa are morphologically very similar to each other and original descriptions are often based on insufficient material (*Grammosciadium haussknechtii*, *Grammosciadium schisckinii* and Grammosciadium pterocarpum subsp. longipes only known from the types, *Grammosciadium armenum* is known from the type and some very few additional individuals).

According to the most recent treatments, *Grammosciadium pterocarpum*, *Grammosciadium haussknechtii* and *Grammosciadium schischkinii* are currently accepted as distinct species and the other taxa of *Grammosciadium armenum* and Grammosciadium pterocarpum subsp. longipes were synonymised under *Grammosciadium pterocarpum* (Vinogradova 1995, Pimenov and Sutory 2014, Bani and Koch 2015).

Numerous specimens were collected from the whole distribution areas, which reflect the morphological and presumably also genetic variation limits of the taxa of section *Stenodiptera* in Turkey for the purpose of a phylogenetic-taxonomic revision of the members of the whole genus *Grammosciadium* between the years of 2011 and 2014. We observed a large number of intermediate forms during our field work. Moreover, we encountered many problems during the identification process of the specimens. The previous diagnostic characters mostly overlapped and some of the populations and specimens were not identified unambiguously. Additionally two populations were discovered recently from Turkey (one is from Sivas province, the other one is from Eskişehir province), and although they are very similar to *Grammosciadium pterocarpum* in terms of their habits, these populations do not match with available species descriptions.

The aims of this study were (1) to examine quantitatively pattern of morphological variation of the members of section *Stenodiptera* based on a representative and population-based sampling with 133 individuals from 17 populations in total, (2) to determine diagnostic morphological and anatomical characters for correct discrimination of the putative taxa, and (3) to provide a taxonomic concept for the newly discovered morphotypes.

## Materials and methods

**Figure 1.**
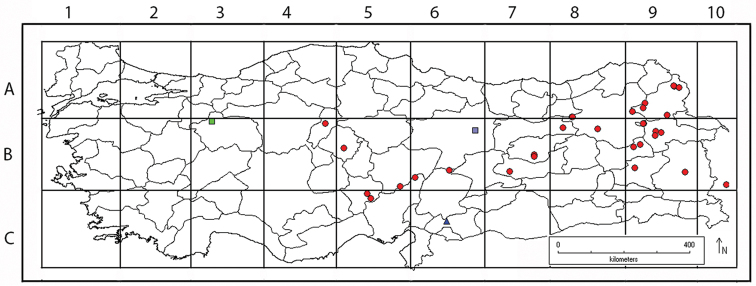
Distribution map of the populations and taxa of section *Stenodiptera* in Turkey analysed herein (Grammosciadium pterocarpum subsp. pterocarpum ●, Grammosciadium pterocarpum subsp. bilgilii ■, Grammosciadium pterocarpum subsp. sivasicum ■, *Grammosciadium haussknechtii* ▴).

133 specimens (individuals) from the members of section *Stenodiptera* were examined and used as operational taxonomic units (OTUs) in the multivariate analyses. *Grammosciadium pterocarpum* (102 individuals from 14 populations), *Grammosciadium haussknechtii* (11 individuals from one population; because it is known from only one locality), and populations of two new subspecies collected from Eskişehir (*B.Bani* 6983) and Sivas (*B.Bani* 6985) provinces of Turkey (10 individuals from one population, respectively). These specimens, which are listed in the Suppl. material 1 and marked with asteriks, include the types of two taxa (*Grammosciadium haussknechtii* and *Grammosciadium schischkinii* see Table 1) and reflected the morphological variability exhibited by the species and populations from throughout its geographic range (Figure 1). Characters used in the multivariate analyses were based on previous taxonomic treatments and our own examination of collected specimens. 12 quantitative characters were selected (Table 1). Characters were scored at the same developmental stage on each plant (fruiting stage). Measurements were taken from the best developed infructescence available on a given specimen. Three data sets were constructed and analyzed: (1) a matrix which included all specimens (133 OTUs and 12 characters), (2) and (3) were created by excluding the OTU’s of *Grammosciadium haussknechtii* and the new subpecies (collected from Eskişehir) from data set 1, respectively. The MANOVA was performed with IBM SPSS Statistics for Windows, Version 20.0. (Armonk, NY), using “Type III sum of squares”, and was followed by “Tukey tests” using the harmonic mean sample size to determine patterns of significant differences between the taxa. The F-test was used to determine which, if any, characters differed significantly among the taxa studied. The PCA was conducted also using IBM SPSS Statistics for Windows, Version 20.0. (Armonk, NY). Identical parameters and procedures were used for all analyses on the three different data sets.

**Table 1.** Morphological characters and their statistics (mean ± standard deviation (SD), min-max range and range with 95% CI: confidence interval) for the four groups (N: number of individuals used for morphological measurements). Characters that differ significantly (P < 0.05) among the taxa as shown by MANOVA are marked with asterisks. Superscript letters indicate the results of Tukey tests, with taxa in the same homogeneous subset (P < 0.05) sharing the same letter.

Characters			*Grammosciadium pterocarpum*			*Grammosciadium haussknechtii* N=11
			subsp. pterocarpum (incl. the type specimen of *Grammosciadium schischkinii* and one accession of *Grammosciadium armenum*) N=102	subsp. sivasicum N=10	subsp. bilgilii N=10	
1	Ray number*	mean± SD min-max 95% CI	9.9±1.7^C^ 6.0–15.0 9.6–10.2	8.5±1.1^BC^ 7.0–11.0 7.4–9.5	6.8±1.3^A^ 5.0–9.0 5.7–7.8	8.2±1.6^AB^ 6.0–10.0 7.2–9.2
2	Ray length (cm)*	mean± SD min-max 95% CI	4.7±1.34^B^ 1.8–9.4 4.5–5.0	3.2±0.86^A^ 2.0–5.0 2.4–4.0	4.2±0.86^AB^ 3.5–5.0 3.4–5.0	4.8±0.73^B^ 3.8–6.2 4.0–5.5
3	Fruiting pedicel length (mm)*	mean± SD min-max 95% CI	5.2±1.37^B^ 3–10 5.0–5.5	3.9±0.92^A^ 2–5 3.1–4.7	4.2±0.42^AB^ 3.5–5.0 3.4–5.0	3.3±1.05^A^ 2–6 2.5–4.1
4	Fruit number*	mean± SD min-max 95% CI	6.7±2.36^A^ 2–13 6.2–7.1	6.3±2.3^A^ 2–10 4.8–7.7	11.4±2.17^B^ 9–13 9.9–12.8	7.9±1.57^A^ 6–11 6.5–9.2
5	Fruit length (cm)*	mean± SD min-max 95% CI	1.10±0.15^B^ 0.7–1.5 1.10–1.16	1.00±0.05^A^ 0.9–1.0 0.9–1.11	0.8±0.94^A^ 0.7–1.1 0.7–0.97	1.3±.15^C^ 1.2–1.7 1.2–1.4
6	Fruit width (mm)*	mean± SD min-max 95% CI	1.0±0.16^A^ 0.8–1.5 1.0–1.1	1.0±0.03^A^ 1.0–1.1 0.9–1.1	1.0±0.00^A^ 1.0–1.0 0.8–1.1	1.32±0.23^B^ 1.0–1.6 1.2–1.4
7	Fruit width/length ratio*	mean± SD min-max 95% CI	1.0±0.18^AB^ 0.6–1.4 1.0–1.1	0.9±0.06^AB^ 0.9–1.0 0.8–1.0	0.8±0.09^A^ 0.7–1.1 0.7–0.9	1.07±0.18^B^ 0.8–1.4 0.9–1.1
8	Fruit wing width (mm)*	mean± SD min-max 95% CI	1.7±0.5^A^ 1–3.1 1.6–1.8	0.85±0.19^B^ 0.5–1.1 0.5–1.1	0.6±0.1^B^ 0.5–0.8 0.3–0.9	0.5±0.0^B^ 0.4–0.6 0.2–0.7
9	Fruit width/wing width ratio*	mean± SD min-max 95% CI	0.6±0.18^A^ 0.3–1.2 0.60–0.61	1.2±0.34^B^ 0.9–2.0 1.0–1.4	1.6±0.31^C^ 1.2–2.0 1.5–1.8	2.6±0.5^D^ 2.0–3.7 2.5–2.8
10	Sepal length (mm)*	mean± SD min-max 95% CI	0.5±0.25^B^ 0.1–1.65 0.5–0.59	0.4±0.12^AB^ 0.1–0.5 0.3–0.67	0.3±0.12^A^ 0.1–0.5 0.1–0.44	0.8±0.20^C^ 0.5–1.0 0.7–1.00
11	Stylopodium length (mm)	mean± SD min-max 95% CI	0.5±0.07^A^ 0.2–0.7 0.51–0.54	0.5±0.03^A^ 0.5–0.6 0.4–0.5	0.5±0.00^A^ 0.5–0.5 0.4–0.5	0.5±0.09^A^ 0.5–0.8 0.50–0.6
12	Style length (mm)	mean± SD min-max 95% CI	0.9±0.15^A^ 0.6–1.50 0.9–0.1	1.0±0.00^A^ 0.9–1.0 1.0–1.0	0.9±0.08^A^ 0.8–1.0 0.8–1.0	1.0±0.03^A^ 1–1.1 1.0–1.0

## Results

### Statistical analysis

Descriptive statistics for the 12 morphological characters are presented in Table 1. The MANOVA showed that 10 characters, except stylopodium and style length, differed significantly (P < 0.05) among the taxa and newly discovered populations, and this was confirmed by Tukey tests (Table 1). Furthermore, only ranges of the characters of sepal length, fruit length, fruit width, Fruit width/wing width ratio, width of fruit wing are not overlapped. Other six characters are overlapped (Table 1).

**Figure 2.**
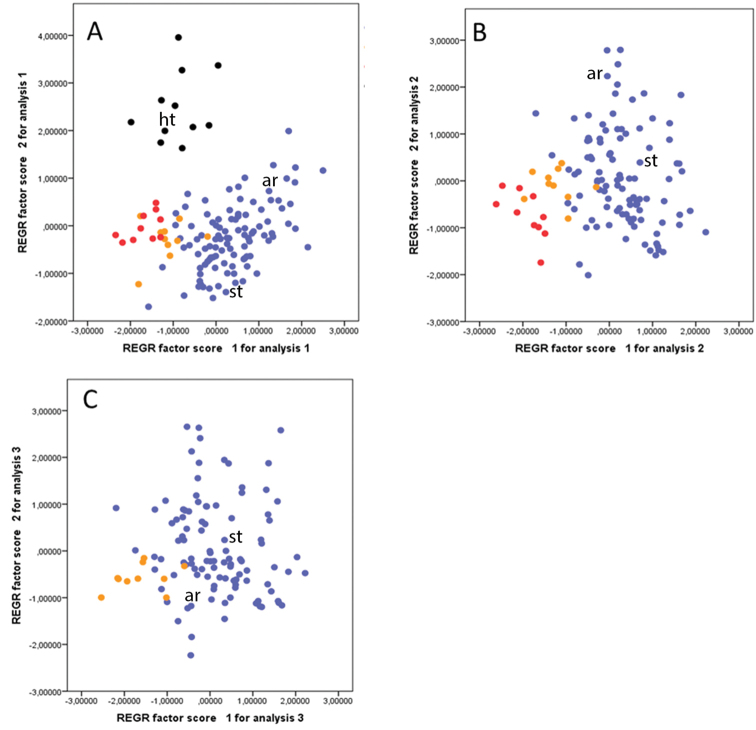
PCA 1-3 with 12 morphological characters. **A** Dataset (1): All the taxa **B** Dataset (2) Grammosciadium pterocarpum subsp. pterocarpum, subsp. bilgilii and subsp. sivasicum **C** Dataset (3) subsp. pterocarpum and subsp. sivasicum. *Grammosciadium haussknechtii* (●), Grammosciadium pterocarpum subsp. pterocarpum (●), subsp. bilgilii (●) and subsp. sivasicum (●) st: type specimen of *Grammosciadium schisckinii*, ar: *Grammosciadium armenum*, ht: type specimen of *Grammosciadium haussknechtii*.

The three Principal Component Analyses of the different datasets, which were performed for OTU’s of *Grammosciadium haussknechtii*, *Grammosciadium pterocarpum*, subsp. bilgilii and subsp. sivasicum with fruiting characters are given in Figure 2 (the first PCA: 12 characters for 133 plots of all the taxa, the second PCA: 12 characters for 122 plots by excluding the plots of *Grammosciadium haussknechtii*, the third PCA: 12 characters for 112 plots by excluding the plots of subsp. bilgilii). The results of the PCA analyses are as follows: the first two components account for a total of (23.78% and 17.82%) 41.60% (dataset 1), (29.90% and 14.90%) 44.80% (dataset 2) and (27.08% and 15.55%) 42.63% (dataset 3) of the variance, respectively. The factor loadings of the first two components for each PCA are given in Table 2.

**Table 2.** Factor loadings for the 12 fruiting characters on the first two components for the 84 OTUs of section *Stenodiptera* members. The values with larger magnitudes are shown in bold for each PC.

	1^st^ PCA		2^nd^ PCA		3^rd^ PCA	
	PC1	PC2	PC1	PC2	PC1	PC2
Sepal length	.286	**.660**	.441	.125	.293	.167
Fruit length	**.550**	.702	**.805**	-.250	**.779**	-.173
Fruit width	.020	.511	.248	.**722**	.129	**.791**
Fruit width/length ratio	**.539**	.206	**.545**	-.766	**.566**	-.768
Fruit width/wing ratio	-.647	**.675**	-.815	-.053	-.747	-.049
Fruits wing width	**.780**	-.367	**.806**	.127	**.752**	.210
Stylopodium length	.182	.028	.213	.021	.148	.058
Style length	.062	-.046	.079	.021	.035	-.002
Fruiting pedicel length	**.634**	-.152	**.580**	.121	**.553**	.285
Ray length	**.710**	.394	**.707**	-.202	**.814**	.060
Fruit number	-.145	.222	-.166	-.414	.308	-.187
Ray number	.433	-292	.396	**.597**	.103	**.650**

### Description of two new subspecies

Based on the morphometric results we can significantly distinguish and characterize the following new subspecies of *Grammosciadium pterocarpum*:

#### Grammosciadium pterocarpum Boiss. subsp. bilgilii

Taxon classificationPlantaeApialesApiaceae

B.Bani subsp. n.

urn:lsid:ipni.org:names:60472798-2

Figs 3 , 4 , 5 , 6

##### Diagnosis.

The new subspecies is similar to Grammosciadium pterocarpum subsp. pterocarpum, but mainly differs from it by the fruits with the narrower wings of lateral ribs (0.5–0.8 mm, not 1–3.1 mm).

##### Type.

Turkey. B3 Eskişehir: around of Yarımca village, clearings of oak woodland, 1250 m, 20.06.2014, *B.Bani 6983*, *E.D.Güner* (holotype GAZI!).

Perennial, erect, branched (at third node or above) herbs. Rootstock with remaining of elder leaf bases. Stem 35–50 cm long and 0.15–0.40 mm broad (just below the first node), angular, prominently or slightly striate, always smooth, white, green or purplish at base. Basal leaves petiolate; petioles 2.5-8 cm long, broadly sheathed towards base, always smooth, prominently ribbed, canaliculate or flat, angular or triangular. Lamina 5–pinnatisect, 5.5–12 cm long, glabrous, linear-elliptic in outline; primary segments 0.5-1.3 cm long, distance between primary segments 0.4–1.5 cm long; ultimate segments 2–4 mm long, mucronate at apex. Lower leaf sheaths mostly connate at base, with stipular segments at margins; stipular segments sessile or shortly petiolate. Upper leaves similar but decreasing in size upwards. Bracts 3-6, trisect or up to 1–3–pinnatisect, 0.6–1.6 cm long; rarely narrowly sheathed; segments 0.3-0.7 cm, always smooth, mucronate at apex. Rays 5–9, unequal, 2.5–5.5 cm. Bracteoles 5-7, trisect to 1–pinnatisect, 0.35-0.65 cm long, always smooth. Flowers male only or hermaphrodite, 8-18, slightly radiate. Pedicels of male flowers 0.15–0.6 cm long. Sepals 0.14–0.5 mm long, smooth, patent or erect. Petals cordate, with long central oil duct, largest petal 2.8–3.5 mm long. Stamens 5; longest filament 1.3–2 mm long. Fruiting pedicels 3.5–5 mm long. Fruits, 9–16 per umbellule, oblong or narrowly lanceolate, 0.7–1.1 × 0.1 cm; each mericarp has 5 primary ribs and four secondary ribs alternating with the primary ribs; lateral ribs winged; wings 0.5–0.8 mm. Stylopodium minute up to 0.5 mm long. Styles divergent, 0.8–1 mm, uninerved on outer side. Flowering May–June; fruiting June–July.

##### Etymology.

We dedicate this new subspecies in memory of our dear colleague Dr. Bilgehan Bilgili who passed away in 2015.

**Figure 3.**
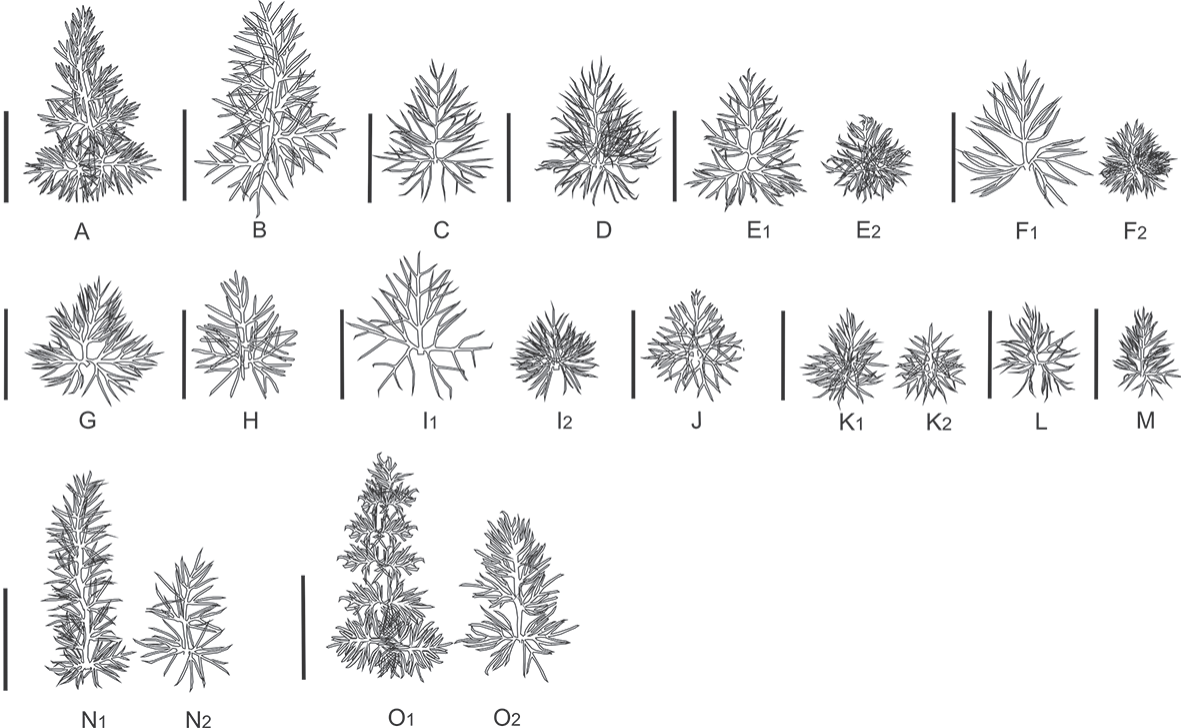
Primer segments of basal leaves in the section *Stenodiptera*. **A–M** Grammosciadium pterocarpum subsp. pterocarpum (**A** *B.Bani* 6966 **D** *B.Bani* 6825 **E** *B.Bani* 6820 **F** *B.Bani* 6997 **G** *B.Bani* 6999 **H** *B.Bani* 6994 **I** *B.Bani* 6931 **J** *B.Bani* 6977 **K** *B.Bani* 6976 **L** *B.Bani* 6932 **M** *B.Bani* 6926) **B** subsp. bilgilii (*B.Bani* 6983) **C** subsp. sivasicum (*B.Bani* 6985) **N** *Grammosciadium haussknechtii* (*B.Bani* 6903) **O** *Grammosciadium platycarpum* (*B.Bani* 6810). Scale bar represents 5 mm.

**Figure 4.**
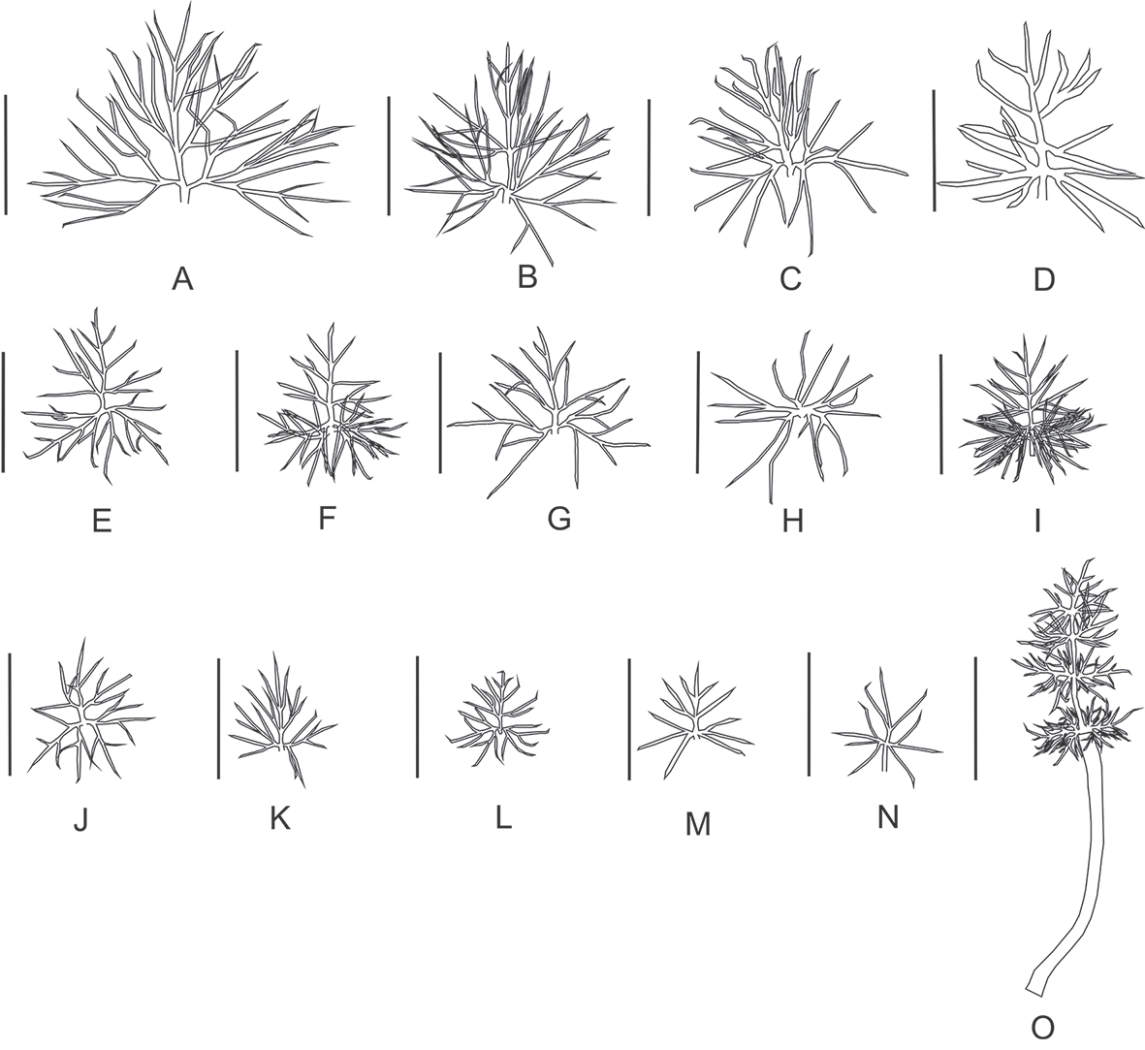
Stipular segments of lower leaf sheaths in the section *Stenodiptera*. **A–M** Grammosciadium pterocarpum subsp. pterocarpum (**A** *B.Bani* 6969 **B** *B.Bani* 6932 **D** *B.Bani* 6966 **E** *B.Bani* 6825 **F** *B.Bani* 6820 **G** *B.Bani* 6977 **H** *B.Bani* 6994 **J** *B.Bani* 6912 **K** *B.Bani* 6926 **L** *B.Bani* 6931 **M** *B.Bani* 6997) **C** subsp. bilgilii (*B.Bani* 6983) **I** subsp. sivasicum (B.B.6985) **N** *Grammosciadium haussknechtii* (*B.Bani* 6903), O: *Grammosciadium platycarpum* (*B.Bani* 6810). Scale bar represents 5 mm.

**Figure 5.**
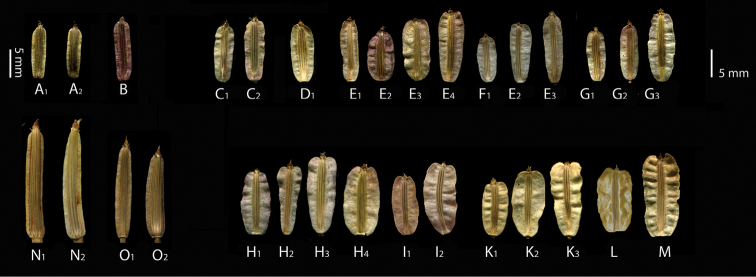
Fruit variations in the section *Stenodiptera* members. **A** Grammosciadium pterocarpum subsp. bilgilii (*B.Bani* 6983) **B** Grammosciadium pterocarpum subsp. sivasicum (*B.Bani* 6985) **C–M** subsp. pterocarpum (**C** *B.Bani* 6969 **D** *B.Bani* 6926 **E** *B.Bani* 6932 **F** *B.Bani* 6999 **G** *B.Bani* 6994 **H** *B.Bani* 6977 **I** *B.Bani* 6885 **K** *B.Bani* 6931 **L** Type of *Grammosciadium schischkinii* **M** *B.Bani* 6872) **N** *Grammosciadium platycarpum* (*B.Bani* 6850) **O** *Grammosciadium haussknechtii* (*B.Bani* 6903).

**Figure 6.**
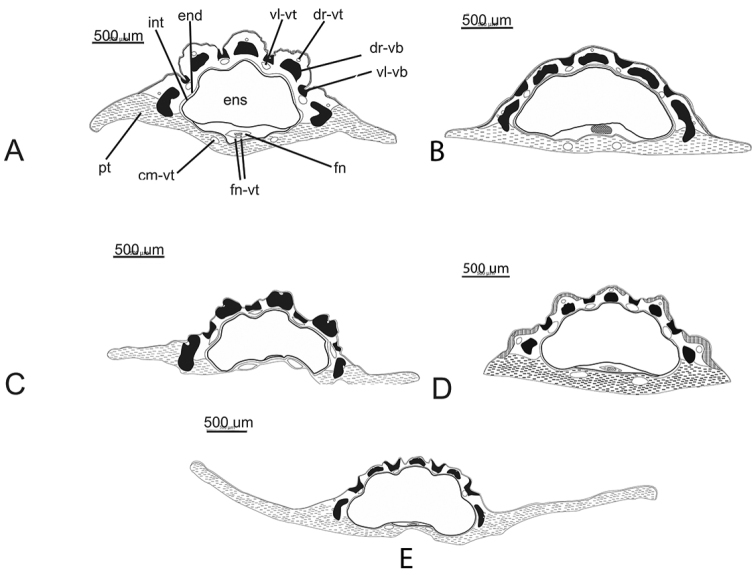
Transvers section of mericarps. **A** Grammosciadium pterocarpum subsp. sivasicum (*B.Bani* 6985) **B** *Grammosciadium haussknechtii* (*B.Bani* 6903) **C** Grammosciadium pterocarpum subsp. bilgilii (*B.Bani* 6983) **D** *Grammosciadium platycarpum* (*B.Bani* 6810) **E** Grammosciadium pterocarpum subsp. pterocarpum (*B.Bani* 6932). cm-vt: commissural vittae. dr-vb: dorsal vascular bundle. dr-vt: dorsal vittae. end: endepidermis. ens: endosperm. fn: funiculus. fn-vt: funicular vittae. int: integument. pt: pterenchyma. vl-vb: vallecular vascular bundle. vl-vt: vallecular vittae.

#### Grammosciadium pterocarpum Boiss. subsp. sivasicum

Taxon classificationPlantaeApialesApiaceae

B.Bani subsp. n.

urn:lsid:ipni.org:names:60472799-2

Figs 3 , 4 , 5 , 6

##### Diagnosis.

The new subspecies is similar to Grammosciadium pterocarpum subsp. pterocarpum and *Grammosciadium platycarpum*, but mainly differs from Grammosciadium pterocarpum subsp.pterocarpum by the fruits with two oil ducts in funiculus, and it differs from *Grammosciadium platycarpum* by its shorter fruits (0.9–1.1 cm, not 1.2–1.8 cm) and lower leaf sheaths without distinctly stalked stipular segments.

##### Type.

Turkey. B6 Sivas: Zara, around of Taşgöze village, steppe, 1920 m, 07.07.2014, *B.Bani 6985*, *M.A.Karakaya* (holotype GAZI!).

Perennial, erect, branched (at third node or above) or unbranched herbs. Rootstock with remaining of elder leaf bases. Stem 23–46 cm long and 0.2–0.4 mm broad (just below the first node), angular, prominently or slightly striate, scabrid or smooth, white, green or purplish at base. Basal leaves petiolate; petioles 6.5–11.5 cm long, broadly sheathed towards base; always smooth, prominently ribbed, canaliculate or flat, angular or triangular. Lamina 4-5–pinnatisect, 5.5–12 cm long, glabrous, glabrous, linear-elliptic in outline; primary segments 0.4–1 cm long, distance between primary segments 0.7–1.3 cm long; ultimate segments 3–5 mm long, mucronate at apex. Upper leaves similar but decreasing in size upwards. Bracts 3–6, trisect or up to 1–2 pinnatisect, (0.7–2.3 cm long; never with hyaline margin; segments 0.3–0.75 cm, always glabrous, mucronate at apex. Rays 7–11, unequal, 2–5 cm. Bracteoles 5–8, mostly simple and rarely trisect or 1-pinnatisect, 0.3–1 cm long, always glabrous. Flowers male only or hermaphrodite, 8–14, slightly radiate. Pedicels of male flowers 0.3–0.5 cm long. Sepals 0.1–0.5 mm long, smooth, patent or erect. Petals cordate, with long central oil duct, largest petal to 3 mm long. Stamens 5; longest filament to 1.5 mm long. Fruiting pedicels 2–5 mm long. Fruits, 2–10 per umbellule, linear-oblong, 0.9–1.1×0.1–0.11 cm long; each mericarp has 5 primary ribs and four secondary ribs alternating with the primary ribs; lateral ribs winged; wings 0.5-1.1 mm. Stylopodium minute up to 0.6 mm long. Styles divergent, ca. 1 mm, uninerved on outer side. Flowering May–June; fruiting June–July.

##### Etymology.

The epithet of this new subspecies derived from Sivas province of Turkey where this taxon is distributed.

Primary leaf segments of basal leaves are shown in Figure 3. *Grammosciadium haussknechtii* is distinctly different with the linear oblong leaf segments. *Grammosciadium platycarpum*, Grammosciadium pterocarpum subsp. bilgilii and one population of Grammosciadium pterocarpum subsp. pterocarpum (*B.Bani* 6966) have lanceolate primary segments. The others do have ovate-lanceolate to orbicular leaf shapes. Stipular segments which are shown in Figure 4 are quite similar each other except stipular segments of *Grammosciadium platycarpum* which are long pedicellate. As shown in Figure 5, the fruits are ordered from narrowly winged to broadly winged one. The width of fruit wings is very highly variable character. It is impossible to distinguish *Grammosciadium schischkinii* with wider fruit wings which was previously used as diagnostic character. This species has clearly similar fruits with fruits of subsp. pterocarpum. *Grammosciadium haussknechtii*, *Grammosciadium platycarpum* and Grammosciadium pterocarpum subsp. bilgilii and subsp. sivasicum have relatively narrow winged fruits than the fruits of subsp. pterocarpum. Figure 4 present the fruit anatomical structure of all taxa in the section *Stenodiptera*. *Grammosciadium platycarpum* and Grammosciadium pterocarpum subsp. sivasicum share similar character of presence of two funicular oil ducts in transverse section of mericarps (Figure 6).

## Discussion

Historically the species have been distinguished by a combination of quantitative and qualitative characters (Boissier 1844, 1872, Freyn 1901, Bordzilowski 1915, Vinogradova and Tamamschian 1968, Tamamschian and Vinogradova 1969, 1969a, 1970, Vinogradova 1995, Bani and Koch 2015, Bani et al. 2016).

Boissier (1844) described *Grammosciadium pterocarpum* with the following diagnosis based on Aucher’s specimen (with immature fruits) collected from Turkey: stem 8-13 cm, fibrous collar present at base, leaves 4.5×0.6 cm and resembling the leaves of *Carum verticillatum* W.D.J.Koch. Fruits are nearly 1 cm long, and fruit wings are 1.5 mm broad. *Grammosciadium haussknechtii*: stems are 30 cm long, leaves are 8 cm long and less than 0.4 cm broad, fruits are 1–1,2 cm long. *Grammosciadium haussknechtii* is close to *Grammosciadium pterocarpum*, but it differs by its narrower leaves, narrower wings of fruits and shorter calyx teeth (Boissier 1872). This species known only from type material located with various herbaria (WU, E, K, LE). Grammosciadium pterocarpum subsp. longipes was described by Frey in 1901 based on the specimens which were collected by Kronenburg from Van province in Turkey in 1889 (Freyn 1901). The lectotype was designated by Pimenov and Sutory (2014) from the herbaria of BRNM (lectotype) and WU (isolectotype). The diagnostic characters are as follows: large pointed calyx teeth and longer pedicels (these specimens have longer pedicels in contrast to the other specimens of *Grammosciadium pterocarpum*) (Freyn 1901). This subspecies is currently under synonymy of *Grammosciadium pterocarpum* (Pimenov and Sutory 2014). According to Bordzilowski (1915) in its original diagnosis *Grammosciadium armenum* is close to *Grammosciadium pterocarpum* and *Grammosciadium haussknechtii*. It differs from both, by its broader leaves, shorter fruits and marginate stylopodium. It slightly differs from *Grammosciadium pterocarpum* by larger stature, rotundate fruit apex (not truncate) and narrower wings of mericarps, and it differs from *Grammosciadium haussknechtii* in having broader wings of fruits. The type of this species is deposited in KW and few vouchers are fund in LE herbarium. *Grammosciadium schischkinii* is close to *Grammosciadium pterocarpum* but differs from it in having more undulated and broader fruit wings (2.5–3.5 mm not 1.5–2 mm), fewer number of fruits (1–4 compared to 4–9), and a more branched stem (Vinogadova 1995). This species has been described based on one specimen only and is kept in LE herbarium. There is no any other collection.

Although the characters of fruit number, width of fruit wings and undulation of wings have been used previously to distinguish *Grammosciadium schischkinii* from the other species (Vinogradova 1995), our data demonstrates that the measurements obtained from the type specimen of *Grammosciadium schischkinii* clearly overlapped with characters of Grammosciadium pterocarpum subsp. pterocarpum (Table 1). Also undulation of fruit wings is common in nearly all populations. We also achieved similar results for *Grammosciadium armenum*, which has been previously recognized as synonym of *Grammosciadium pterocarpum*. We did not find any qualitative or quantitative character to distinguish these species. The type of Grammosciadium pterocarpum subsp. longipes is also identical with subsp. pterocarpum. Length of calyx teeth and length of pedicels, which has been used as diagnostic characters (Freyn 1901) are overlapping with the other taxa and do not allow reliable differentiation. It is obvious from this study that sufficient fruiting and flowering material is needed for its proper taxonomic treatment.

MANOVA demonstrated that most of the characters differ statistically among the groups (Table 1), and the range values of the various characters (sepal length, fruit length, fruit width, fruit width/wing ratio and width of fruit wings) can be used to distinguish the various taxa significantly. Fruit length and sepal length separate *Grammosciadium haussknechtii* from subsp. sivasicum and subsp. bilgilii. Fruit width is distinguishing between *Grammosciadium haussknechtii* and subsp. bilgilii. *Grammosciadium haussknechtii* is clearly different from the all others by its higher Fruit width/wing width ratio also separates subsp. bilgilii from subsp. pterocarpum. Another diagnostic character is width of fruit wings, which discriminates subsp. pterocarpum from all other taxa.

According to PCA on dataset 1 (complete dataset), individuals of *Grammosciadium haussknechtii* are clearly distinguished from all other taxa as a distinct group mostly because of their larger ratio of fruit width/wing width (2-3,7 mm not 0,3-2 mm). Fruit length, fruit width/length ratio, fruit width/wing ratio, fruiting pedicel and ray length are the most discriminative characters with the largest eigenvalues (Figure 2A, Table 2). With PCA on dataset 2 (subsp. pterocarpum, subsp. sivasicum and subsp. bilgilii) individuals of subsp. bilgilii, are clearly distinguished from subsp. pterocarpum by the narrower wings of fruit (0.5-0.8 mm, not 1-3.1 mm). However, individuals of subsp. sivasicum are placed with an intermediate position between these two groups (Figure 2B). Fruit length, fruit width/length ratio, width of fruit wing, fruiting pedicel, ray length, fruit number and ray number are the characters with highest eigenvalues (Table 2). Similarly, the PCA on the third dataset (subsp. pterocarpum and subsp. sivasicum) separates both taxa from each other, but few individuals of both groups are overlapping (Figure 2C). PCA on dataset 2 and 3 show the same discriminative characters (except fruit number) (Table 2). But as indicated above, subspecies sivasicum has two funicular oil ducts in the funiculus in transverse section of mericarps resembling a unique character within the section *Stenodiptera*.

### Currently accepted taxa with the synonyms and a key to the members of section *Stenodiptera*

**Table:** 

1	Funicular oil duct present in transverse section of mericarps	**Grammosciadium pterocarpum subsp. sivasicum**
–	Funicular oil duct absent in transverse section of mericarps	**2**
2	Fruit wings more than 1 mm	**Grammosciadium pterocarpum subsp. pterocarpum**
–	Fruit wings less than 1 mm	**3**
3	Fertile part of fruits 0.7–1.1×1 mm	**Grammosciadium pterocarpum subsp. bilgilii**
–	Fertile part of fruits 1.2–1.7×1–1.6 mm	***Grammosciadium haussknechtii***

**1. Grammosciadium pterocarpum Boiss. subsp. pterocarpum** in Ann. Sci. Nat. ser. 3, 2: 68 (1844).

Syn: *Stenodiptera pterocarpa* (Boiss.) Koso-Pol. in Bot. Zhurn. 1–2: 13 (1915)

*Caropodium pterocarpum* (Boiss.) Schischkin in Not. Syst. (Leningrad) 4: 30 (1923) Grammosciadium pterocarpum (Boiss.) subsp. longipes Freyn in Bull. Herb. Boiss. 2(1): 245–289 (1901)

*Stenodiptera armena* Bordz. in Mem. Soc. Nat. Kiev 25(1): 96 (1915)

*Caropodium armenum* (Bordz.) Schischkin in Not. Syst. (Leningrad) 4: 30 (1923)

*Grammosciadium schischkinii* (Vinogr. & Tamamsch) Vinogr. in Bot. Zhurn. (1995) **syn. n.**

Caropodium pterocarpum (Boiss.) Schischkin var. schischkinii Vinogr. & Tamamsch. in Notes R.B.G. Edinb. 28: 203 (1968)

**2. Grammosciadium pterocarpum subsp. bilgilii subsp. n.**

**3. Grammosciadium pterocarpum subsp. sivasicum subsp. n.**

**4. *Grammosciadium haussknechtii* Boiss.** in FI. Or. 2:901 (1872)

Syn: *Stenodiptera haussknechtii* (Boiss.) Koso-Pol., in Bot. Zhurn. (1-2): 13 (1915)

*Caropodium haussknechtii* (Boiss.) Schischkin in Not. Syst. (Leningrad) 4: 30 (1923)

## Supplementary Material

XML Treatment for Grammosciadium pterocarpum Boiss. subsp. bilgilii

XML Treatment for Grammosciadium pterocarpum Boiss. subsp. sivasicum

## Supplementary materials

Supplementary material 1 Herbarium specimens of members of section *Stenodiptera* distributed in Turkey Data type: Specimens data (doc file) This dataset is made available under the Open Database License (http://opendatacommons.org/licenses/odbl/1.0/). The Open Database License (ODbL) is a license agreement intended to allow users to freely share, modify, and use this Dataset while maintaining this same freedom for others, provided that the original source and author(s) are credited.Barış Bani, Fatma Ulusoy, Muhammet Ali Karakaya, Marcus A. Koch
