# Supplementary material for: Taxonomic implications from morphological and anatomical studies in the section Stenodiptera from the genus Grammosciadium (Apiaceae)
Source: PhytoKeys. 2016 Aug 9;(68):73–89. doi: 10.3897/phytokeys.68.9089 (PMC5029132; doi:10.3897/phytokeys.68.9089)
Supplement: Supplementary material 1 — Herbarium specimens of members of section Stenodiptera distributed in Turkey [file phytokeys-068-073-s001.docx]

Appendix

Herbarium specimens of members of section *Stenodiptera* distributed in Turkey

***G. pterocarpum* subsp. *pterocarpum*:**

Turkey A8 Bayburt: Kop Da., Bayburt to Aşkale, 2400 m, *Hub.-Mor.* 15412; 2500 m, 28.07.2013, *B.Bani 6948* (GAZI)*. Erzurum: the pass from the valley river Tortum to Karasu valley, Dumlu Dağ, 08.07.1994, *Pimenov, Kljukov, Hohoyakov* 472 (LE)*. A9 Kars: in jogo Saghanluk in pascius pagi Bardus, 17.06.1908, *T. Roop* (LE)*; reperta est in jugo Saghanluk ad stationem ejustem nominis, 07.07.1910, *T. Roop* (KW: type of *G. armenum*); Kağızman, 30.05.1904, *C.Michaelovsky* (LE); 02.06.1913, *Woronow* 12459 (LE); 03.06.1913, *Woronow* 12457 (LE); 07.05.1914, *Turkevicz* 225 (LE); above mountain, 2800 m, 06.20.1914, *Litvinov* (LE)*; Çengilli village, Mantar düzlüğü district, 2000-2400 m, 10.07.1981, *O.Güneş* (HUB); Sarıkamış, 05.06.1914, *Litvinov* (LE); Sarıkamış-Karaurgan, Handere pass, forest clearings, 2360 m, 20.07.2013, *B.Bani* 6929 (GAZI)*; Arpaçay, Karakale village, Karakayalar, rocky slopes, 2150 m, 21.08.1984, *H.Ocakverdi* 2044 (GAZI); South of Çıldır lake, rocky slopes, 2200 m, 19.06.1984, *M.Vural* 2525 (GAZI). B5 Niğde: Bereketli Maden, 2000m, Siehe312 (E); Kayseri: Bakir Da., above Kisge, nr. Akoluk Y., 1500 m, *Davis 19334* (E)*;* Niğde-Çamardı köyü, Üçkapılı köyü çevresi, step, 1895 m, 08.07.2013, *B.B.6926* (GAZI)*. Kırşehir: Boztepe, Kervansaray mountain, North of Yeşiltepe, forest clearings, 1400 m, 23.06.1995*, E. Hamzaoğlu* *2396* *et al*. (YOZGAT); steppe, 1350 m, 11.06.2014, *B.Bani* 6969 (GAZI)*. B7 Elazığ: Harput, Yarbaşı, in herbidis, 20.05.1889, Sintenis 441 (LE); Baskil, after 2 km from Şahaplı village, oak woodland, 1123 m, 07.07.2012, *B.Bani 6885*(GAZI)*; 02.05.2013**,** *B.Bani6912* (GAZI)*. Tunceli: Pertek-Tunceli, 1100 m, slope in oak scrub, 06.06.1957*, Davis 29154, Hedge* (E); 20 km to Tunceli, oak woodland, 1230 m, 03.05.2013, *B.Bani 6914* (GAZI)*. B8 Erzincan: Erzincan-Tercan, 14 km to Tercan, steppe, 1750 m, 04.06.2012, *B.Bani 6819***;** B9 Erzurum: Horasan-Karaurgan, 5 km to Karaurgan, steppe, 2000 m, 05.06.2012, *B.Bani* 6820 (GAZI)*; 20.07.2013, B.B.6931 (GAZI)*. Ağrı: Bajasetskyi Sandjak in districtu Sandjan in declivibus stepposi, 01.07.1916 *B.Schischkin* (LE: type of *G. schischkinii*)*; Kılıçgedik pass, supalpin 11.05.1916, *Schischkin* (LE); Horasan-Eleşkirt, around of Güneyyaka village, steppe, 1990 m, 05.06.2012, *B.Bani 6823* (GAZI)*; Horasan-Eleşkirt, above Tahir village, Tahir pass, steppe, 2400 m, 20.07.2013, *B.Bani 6932* (GAZI)*; 2 km SW of Hamur, (Murat valley), 1670 m, sloping meadow, 01.06.1966, Davis 44158 (E); steppe, 1625 m 05.06.2012, *B.Bani 6825* (GAZI)*; 04.07.2012, *B.Bani 6872* (GAZI)*. Muş: Malazgirt, N of Kozgölü village, steppe, 1800 m, 23.05.2006, *Behçet (1129), et al.* (VANF); Malazgirt, NE of A.Kıcık, steppe, 1763 m, 12.05.2007, *LFM1763* (VANF). Van: Erek mountain, 12.06.1899, *Kronenburg 135* (WU; BRNU, type of *G. pterocarpum* subsp. *longipes*). Bitlis: Tatvan, Nemrut mountain, 7600 ft., on slopes in crater, 03.07.1954, *Davis 23553,* *Polunin* (E); *Betula* woodland, 2000 m, 05.06.2011, *B.Bani 6743*(GAZI)*. C5 Konya: Ereğli, Aydos mountain, Sayıntaş, marvel rocks, 1700 m, 16.07.1977, *S.Erik 2603* (HUB)

***G.* *pterocarpum* subsp. *bilgili***

B3 Eskişehir: around of Yarımca village, clearings of oak woodland, 1250 m, 20.06.2014, *B.Bani 6983, E.D.Güner* (GAZI)*; Sündiken mountain, under *Quercus*, 1300 m, 19.06.1973, *T.Ekim 865* (E)

***G. pterocarpum* Boiss. subsp. *sivasicum* B.Bani**

B6 Sivas: Zara, around of Taşgöze village, steppe, 1920 m, 07.07.2014, *B.Bani* 6985, *M.A.Karakaya* (GAZI)*.

***G. haussknechtii***

C6 Gaziantep: Suf Da., 1219 m, Haussknecht (K)*; Gaziantep-Nurdağ, yamaçoba village, oak woodland, 1100 m, 01.05.2013, *B.Bani 6903*(GAZI)*; 08.05.2013, *B.Bani 6920* (GAZI)*.
